# Supplementary material for: Evaluation of individual and ensemble probabilistic forecasts of COVID-19 mortality in the United States
Source: Proc Natl Acad Sci U S A. 2022 Apr 8;119(15):e2113561119. doi: 10.1073/pnas.2113561119 (PMC9169655; doi:10.1073/pnas.2113561119)
Supplement: Supplementary File [file pnas.2113561119.sd02.pdf]

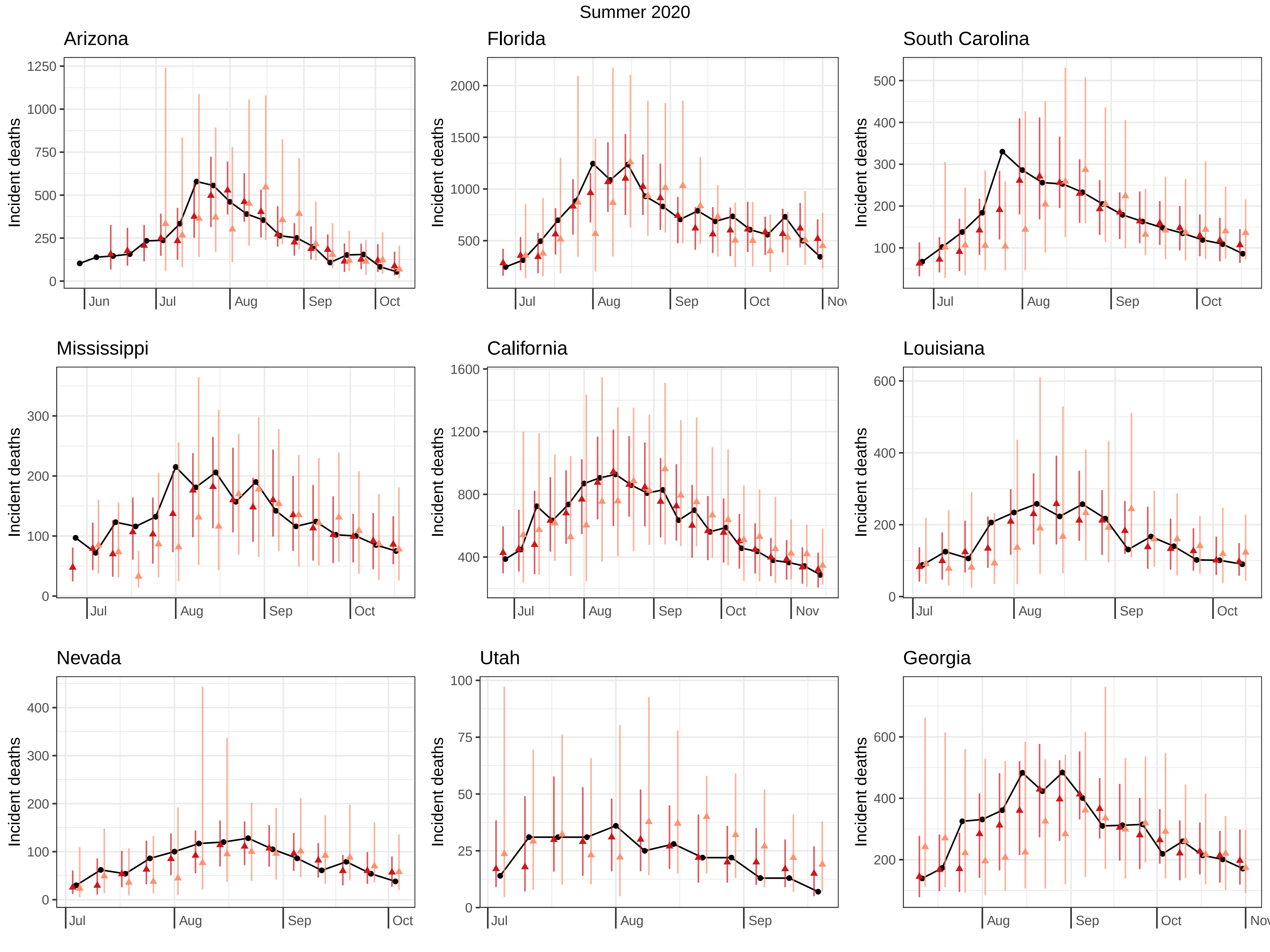

Summer 2020
Arizona
Florida
South Carolina
Mississippi
California
Louisiana
Nevada
Utah
Georgia
Incident deaths
Jun
Jul
Aug
Sep
Oct
Nov

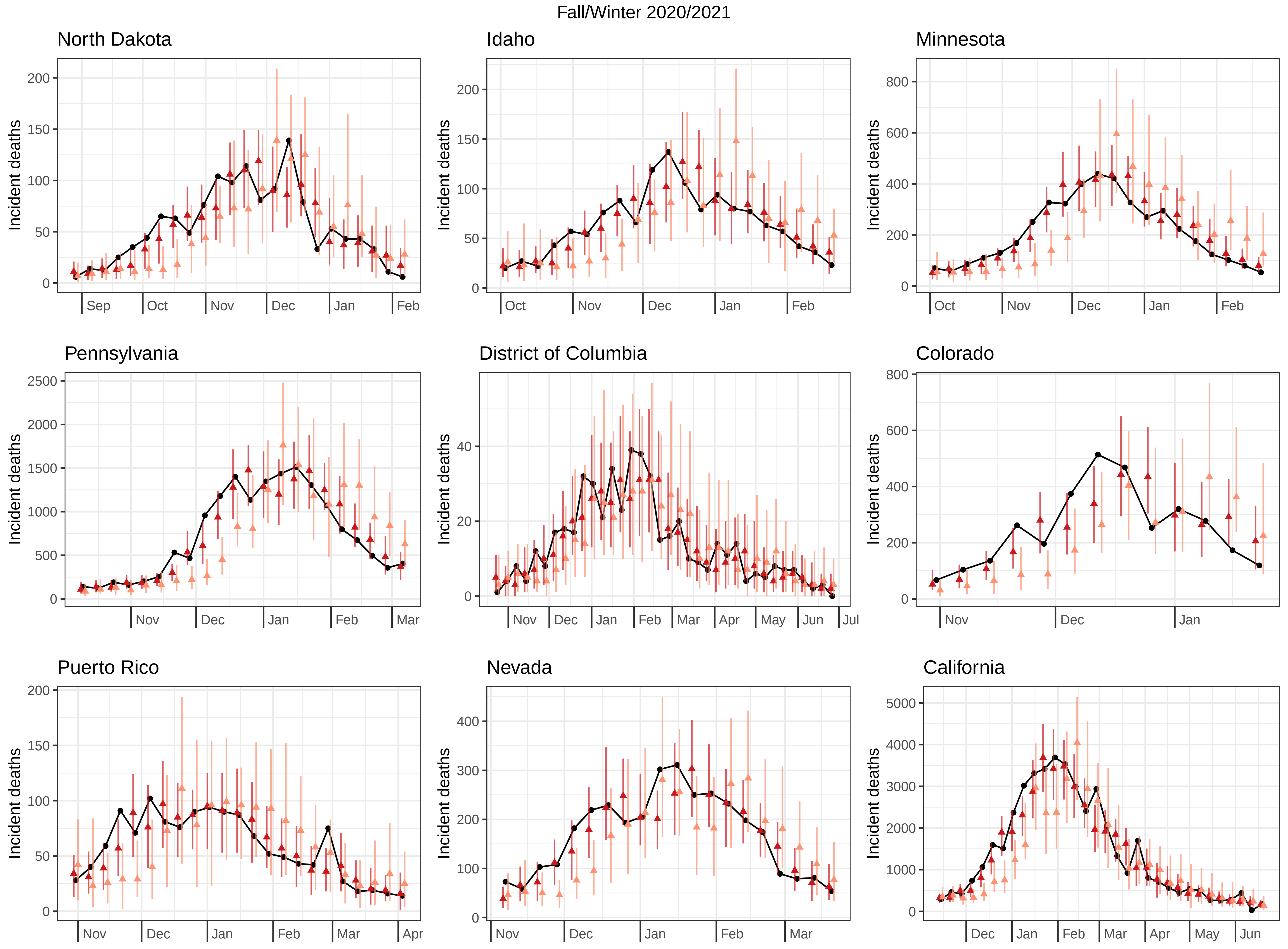

Fall/Winter 2020/2021
North Dakota
Idaho
Minnesota
Pennsylvania
District of Columbia
Colorado
Puerto Rico
Nevada
California
Incident deaths

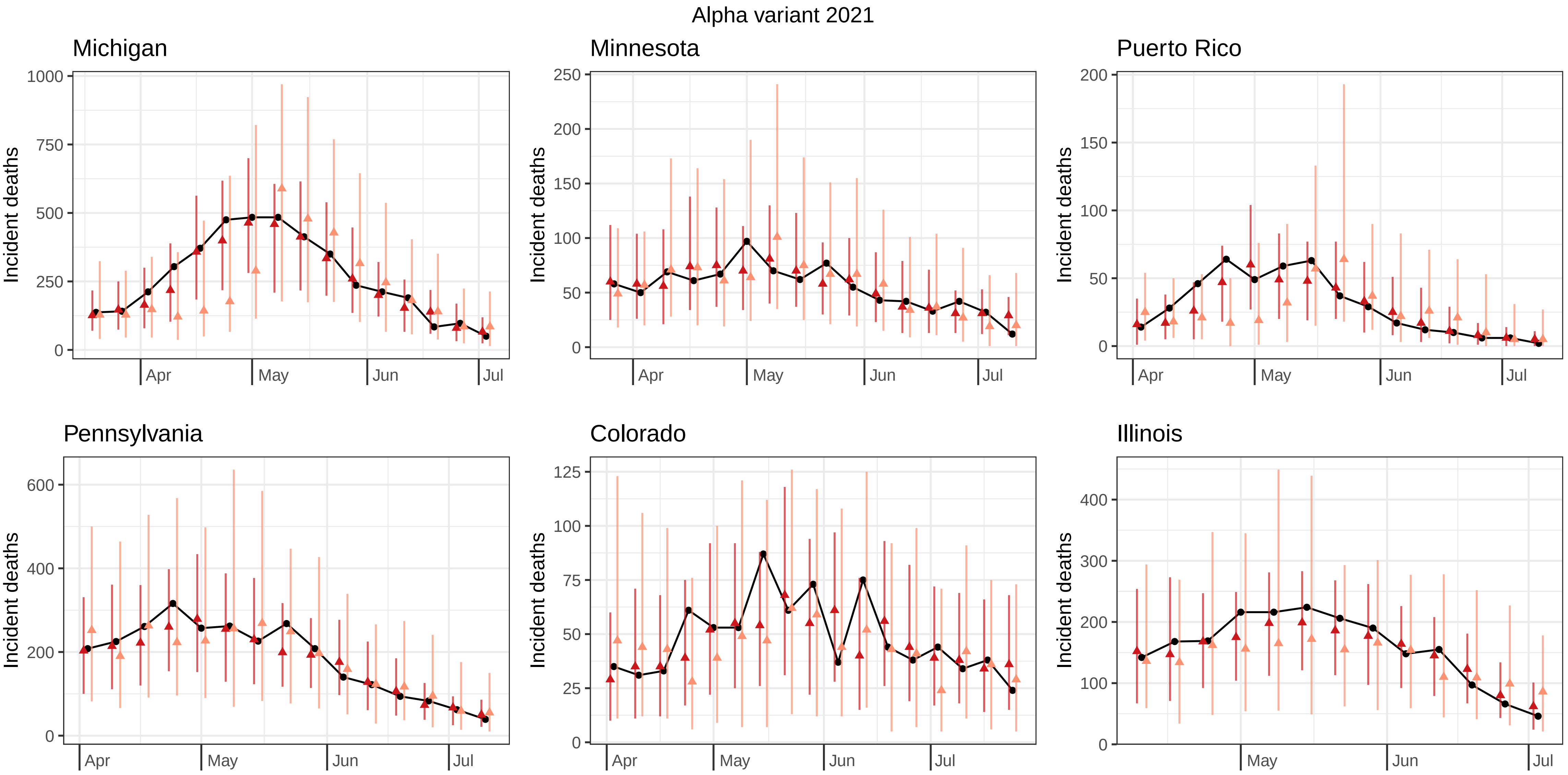

Alpha variant 2021
Michigan
Minnesota
Puerto Rico
Pennsylvania
Colorado
Illinois
Incident deaths
1000
750
500
250
0
250
200
150
100
50
0
200
150
100
50
0
600
400
200
0
125
100
75
50
25
0
400
300
200
100
0
Apr
May
Jun
Jul

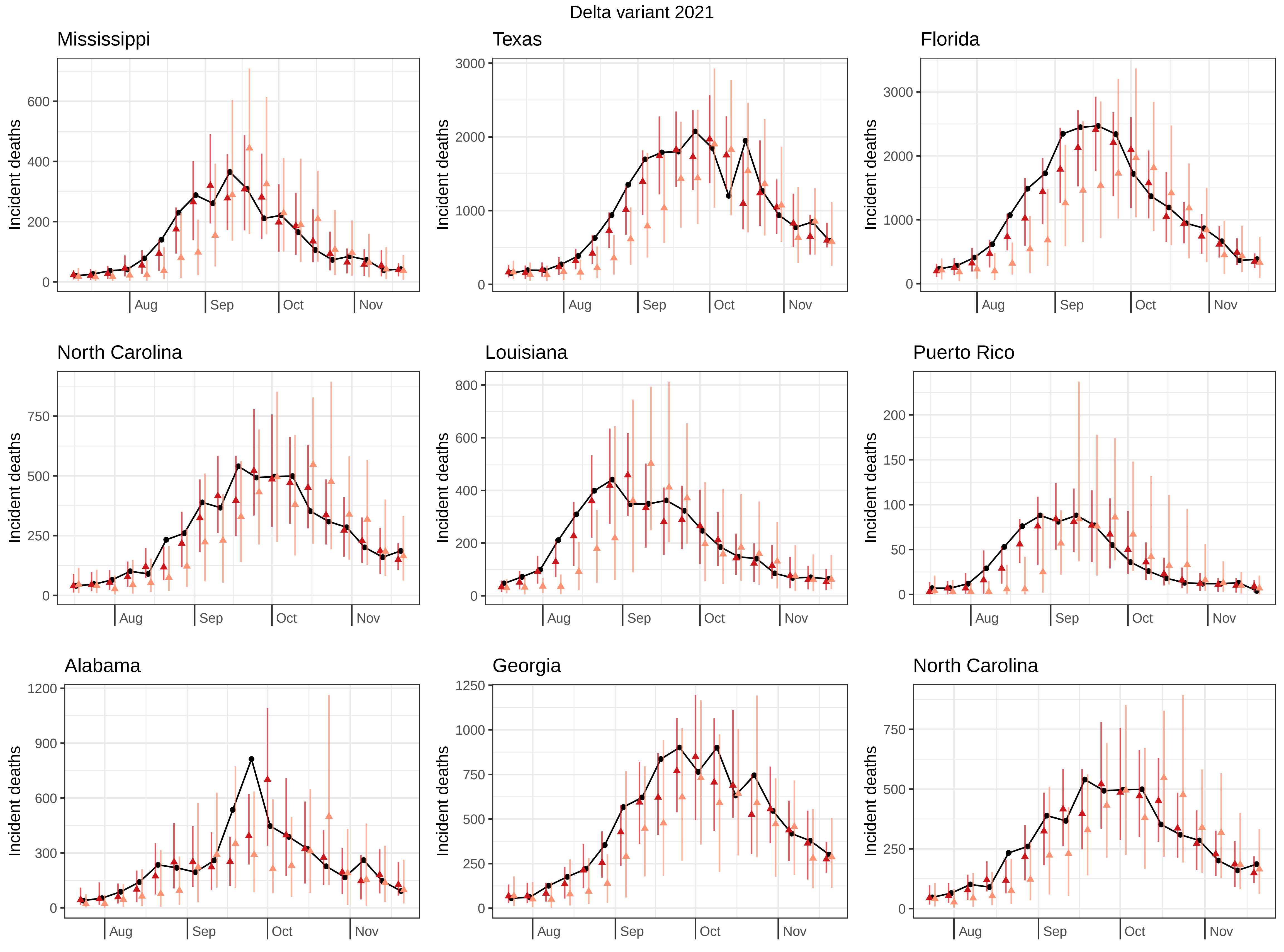
Delta variant 2021
Mississippi
Texas
Florida
North Carolina
Louisiana
Puerto Rico
Alabama
Georgia
North Carolina
Incident deaths
Aug
Sep
Oct
Nov
