# Supplementary figures and images for: Evaluation of individual and ensemble probabilistic forecasts of COVID-19 mortality in the United States
Source: Proc Natl Acad Sci U S A. 2022 Apr 8;119(15):e2113561119. doi: 10.1073/pnas.2113561119 (PMC9169655; doi:10.1073/pnas.2113561119)

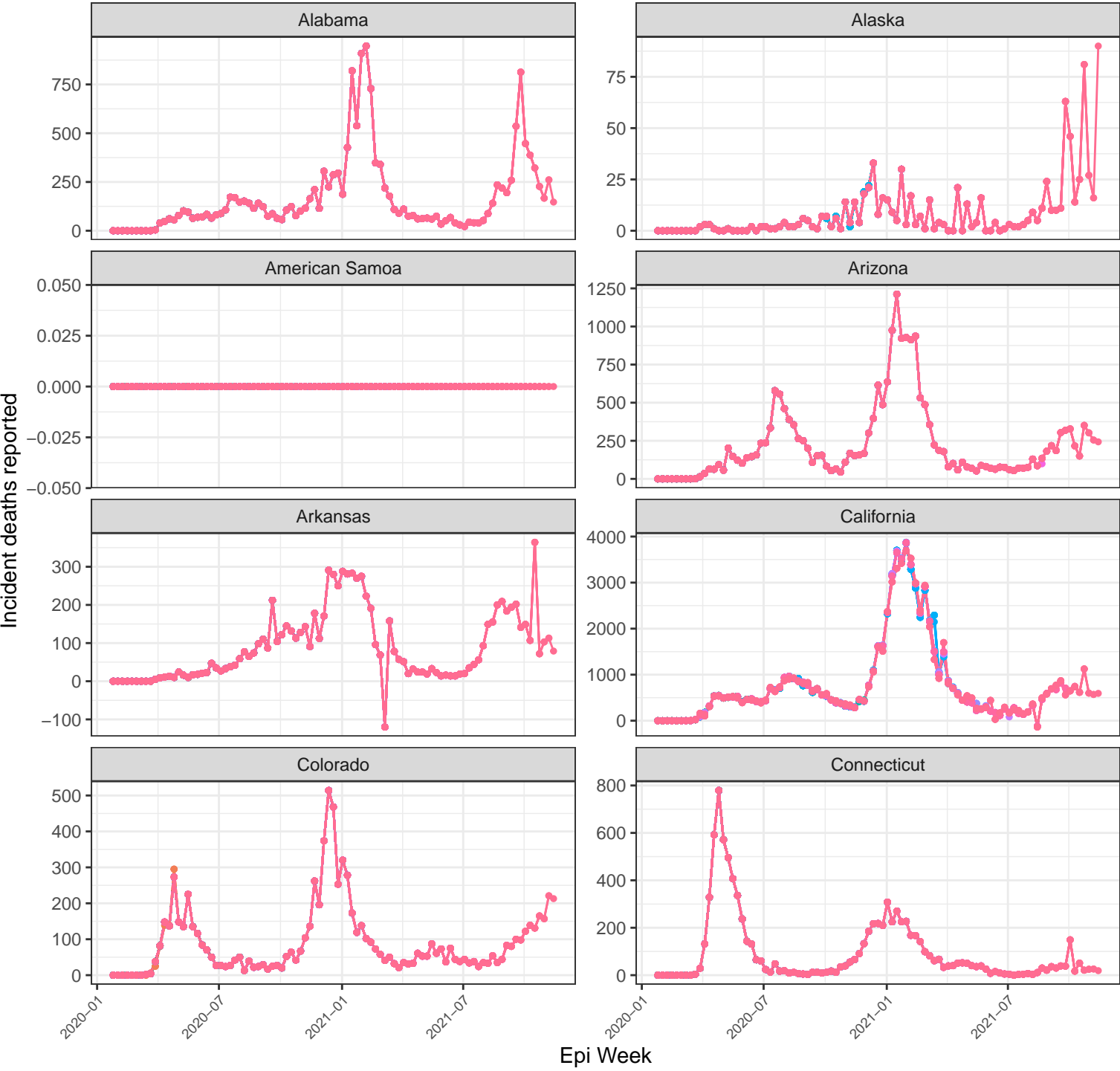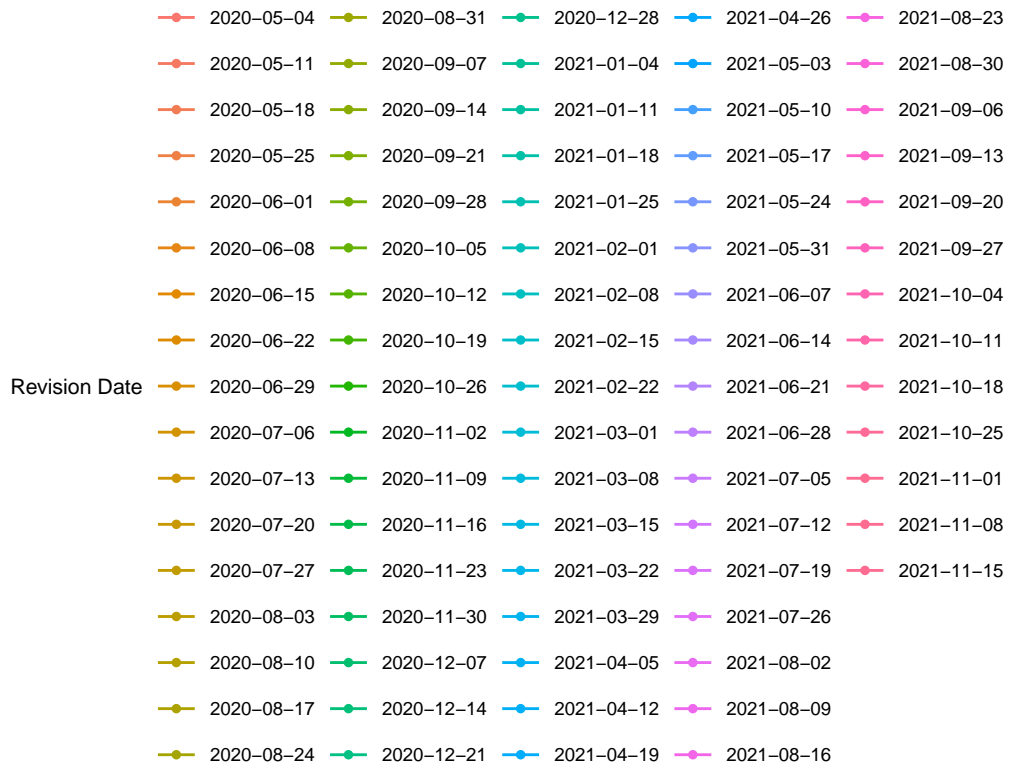

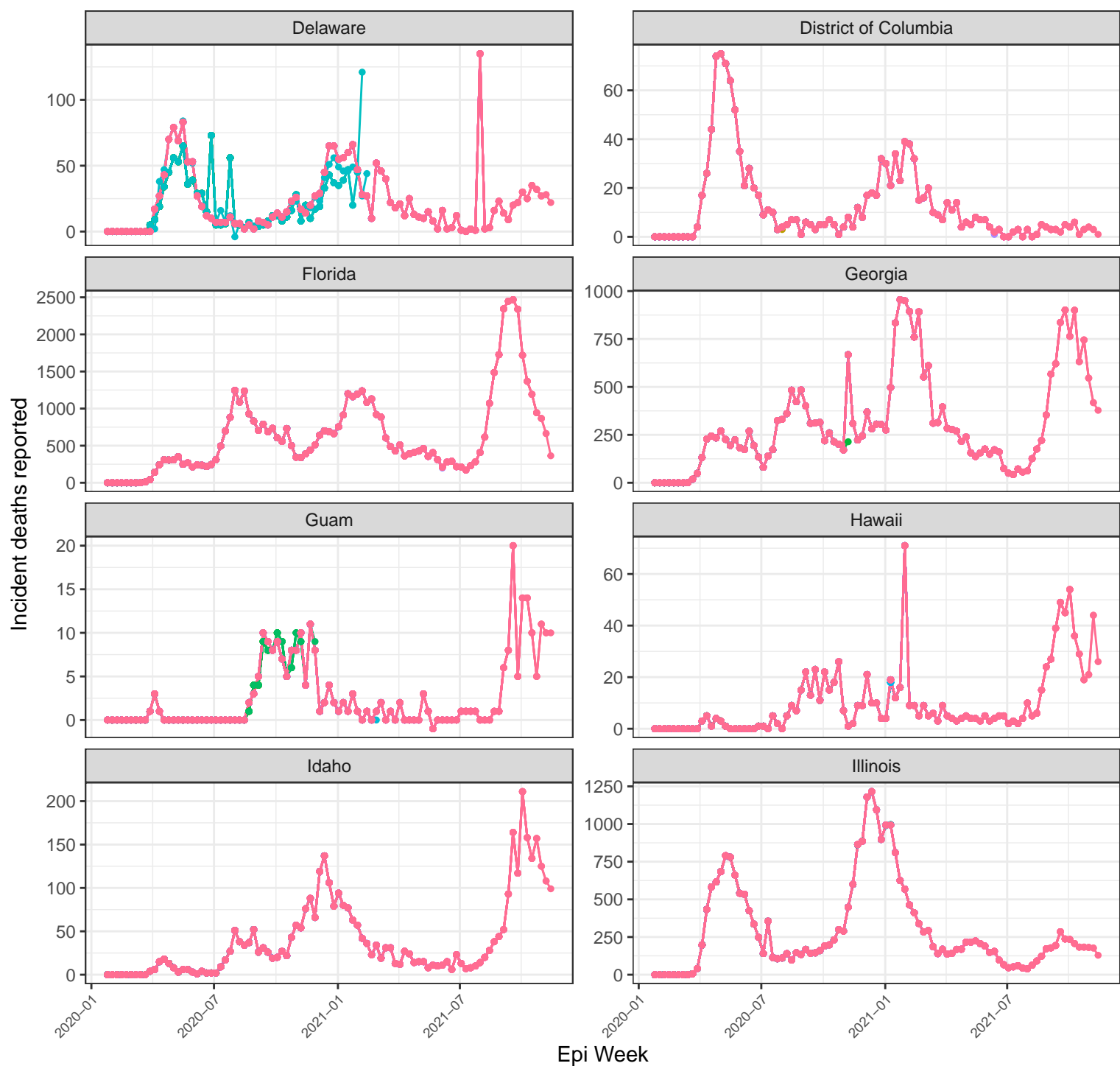

|               |            |            |            |            |            |
|---------------|------------|------------|------------|------------|------------|
|               | 2020-05-04 | 2020-08-31 | 2020-12-28 | 2021-04-26 | 2021-08-23 |
|               | 2020-05-11 | 2020-09-07 | 2021-01-04 | 2021-05-03 | 2021-08-30 |
|               | 2020-05-18 | 2020-09-14 | 2021-01-11 | 2021-05-10 | 2021-09-06 |
|               | 2020-05-25 | 2020-09-21 | 2021-01-18 | 2021-05-17 | 2021-09-13 |
|               | 2020-06-01 | 2020-09-28 | 2021-01-25 | 2021-05-24 | 2021-09-20 |
|               | 2020-06-08 | 2020-10-05 | 2021-02-01 | 2021-05-31 | 2021-09-27 |
|               | 2020-06-15 | 2020-10-12 | 2021-02-08 | 2021-06-07 | 2021-10-04 |
|               | 2020-06-22 | 2020-10-19 | 2021-02-15 | 2021-06-14 | 2021-10-11 |
| Revision Date | 2020-06-29 | 2020-10-26 | 2021-02-22 | 2021-06-21 | 2021-10-18 |
|               | 2020-07-06 | 2020-11-02 | 2021-03-01 | 2021-06-28 | 2021-10-25 |
|               | 2020-07-13 | 2020-11-09 | 2021-03-08 | 2021-07-05 | 2021-11-01 |
|               | 2020-07-20 | 2020-11-16 | 2021-03-15 | 2021-07-12 | 2021-11-08 |
|               | 2020-08-03 | 2020-11-23 | 2021-03-22 | 2021-07-19 | 2021-11-15 |
|               | 2020-08-10 | 2020-12-07 | 2021-04-05 | 2021-08-02 |            |
|               | 2020-08-17 | 2020-12-14 | 2021-04-12 | 2021-08-09 |            |
|               | 2020-08-24 | 2020-12-21 | 2021-04-19 | 2021-08-16 |            |

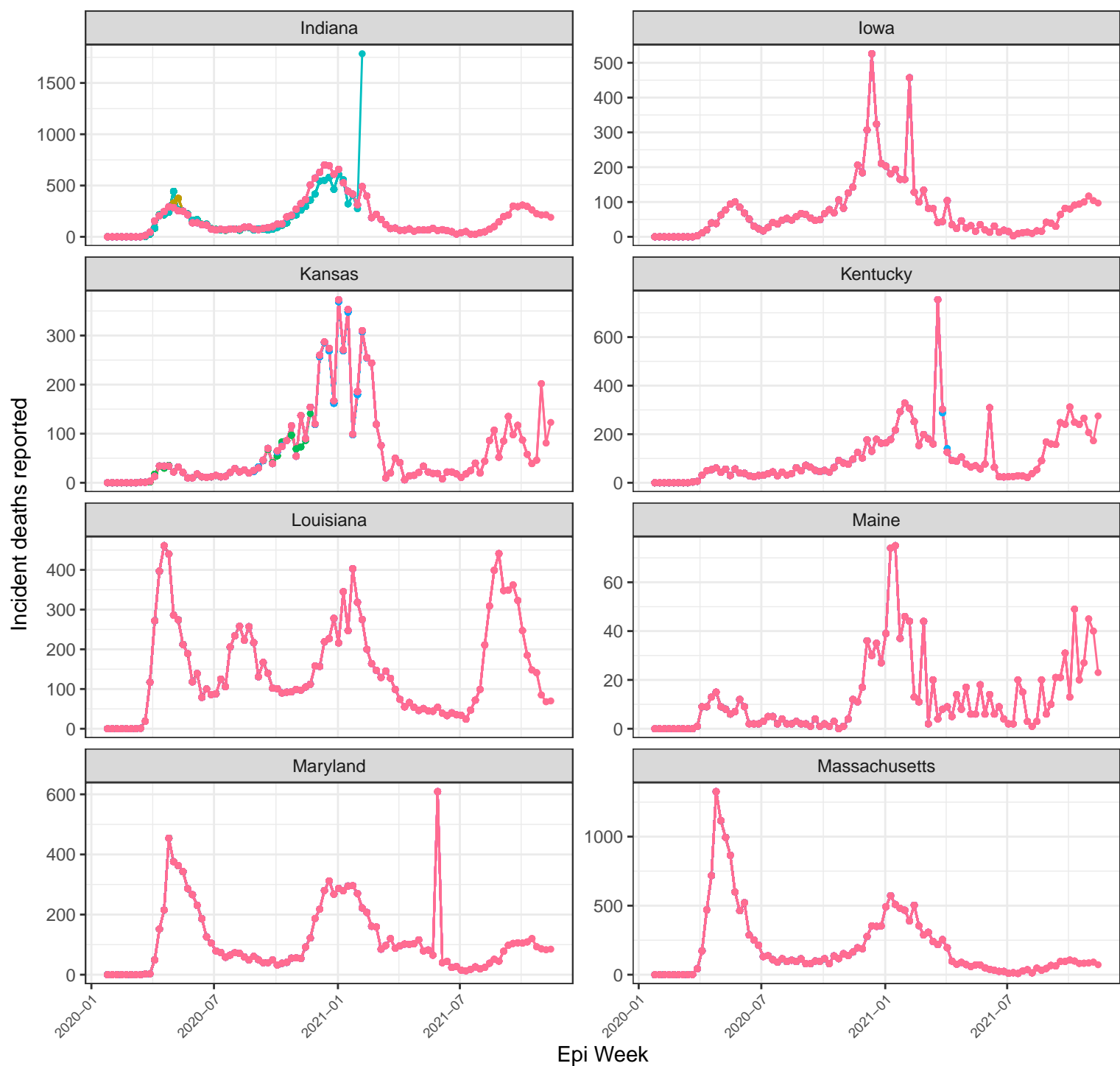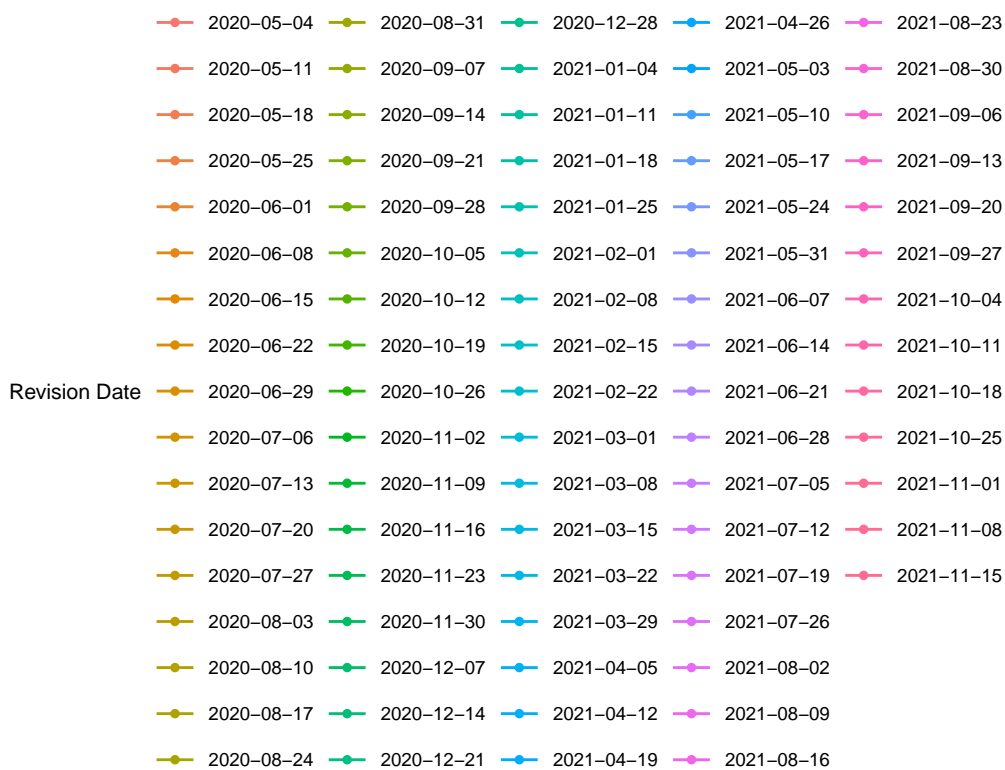

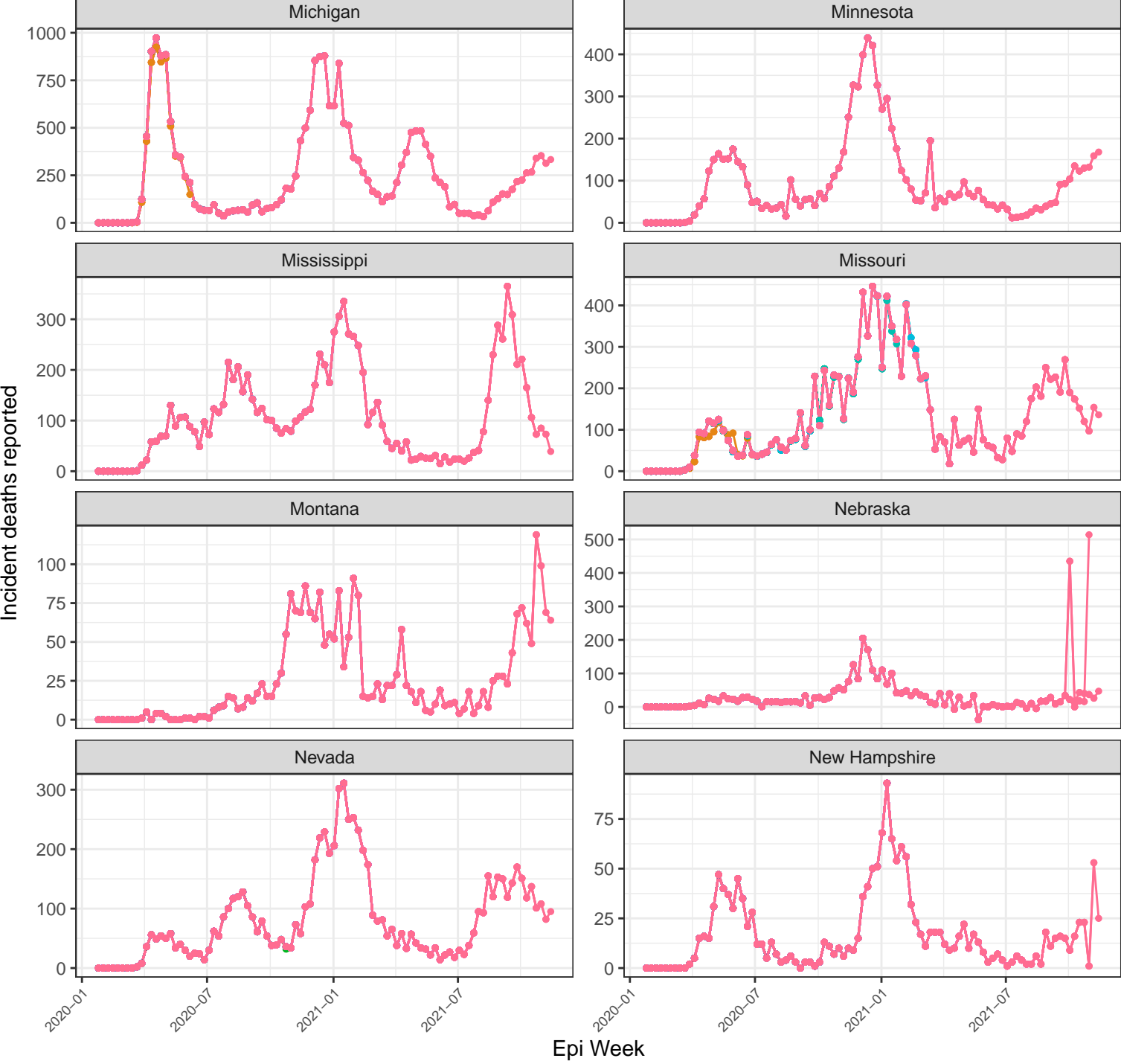

|               |  |            |  |            |  |            |  |            |  |            |
|---------------|--|------------|--|------------|--|------------|--|------------|--|------------|
| Revision Date |  | 2020-05-04 |  | 2020-08-31 |  | 2020-12-28 |  | 2021-04-26 |  | 2021-08-23 |
|               |  | 2020-05-11 |  | 2020-09-07 |  | 2021-01-04 |  | 2021-05-03 |  | 2021-08-30 |
|               |  | 2020-05-18 |  | 2020-09-14 |  | 2021-01-11 |  | 2021-05-10 |  | 2021-09-06 |
|               |  | 2020-05-25 |  | 2020-09-21 |  | 2021-01-18 |  | 2021-05-17 |  | 2021-09-13 |
|               |  | 2020-06-01 |  | 2020-09-28 |  | 2021-01-25 |  | 2021-05-24 |  | 2021-09-20 |
|               |  | 2020-06-08 |  | 2020-10-05 |  | 2021-02-01 |  | 2021-05-31 |  | 2021-09-27 |
|               |  | 2020-06-15 |  | 2020-10-12 |  | 2021-02-08 |  | 2021-06-07 |  | 2021-10-04 |
|               |  | 2020-06-22 |  | 2020-10-19 |  | 2021-02-15 |  | 2021-06-14 |  | 2021-10-11 |
|               |  | 2020-06-29 |  | 2020-10-26 |  | 2021-02-22 |  | 2021-06-21 |  | 2021-10-18 |
|               |  | 2020-07-06 |  | 2020-11-02 |  | 2021-03-01 |  | 2021-06-28 |  | 2021-10-25 |
|               |  | 2020-07-13 |  | 2020-11-09 |  | 2021-03-08 |  | 2021-07-05 |  | 2021-11-01 |
|               |  | 2020-07-20 |  | 2020-11-16 |  | 2021-03-15 |  | 2021-07-12 |  | 2021-11-08 |
|               |  | 2020-07-27 |  | 2020-11-23 |  | 2021-03-22 |  | 2021-07-19 |  | 2021-11-15 |
|               |  | 2020-08-03 |  | 2020-11-30 |  | 2021-03-29 |  | 2021-07-26 |  |            |
|               |  | 2020-08-10 |  | 2020-12-07 |  | 2021-04-05 |  | 2021-08-02 |  |            |
|               |  | 2020-08-17 |  | 2020-12-14 |  | 2021-04-12 |  | 2021-08-09 |  |            |
|               |  | 2020-08-24 |  | 2020-12-21 |  | 2021-04-19 |  | 2021-08-16 |  |            |

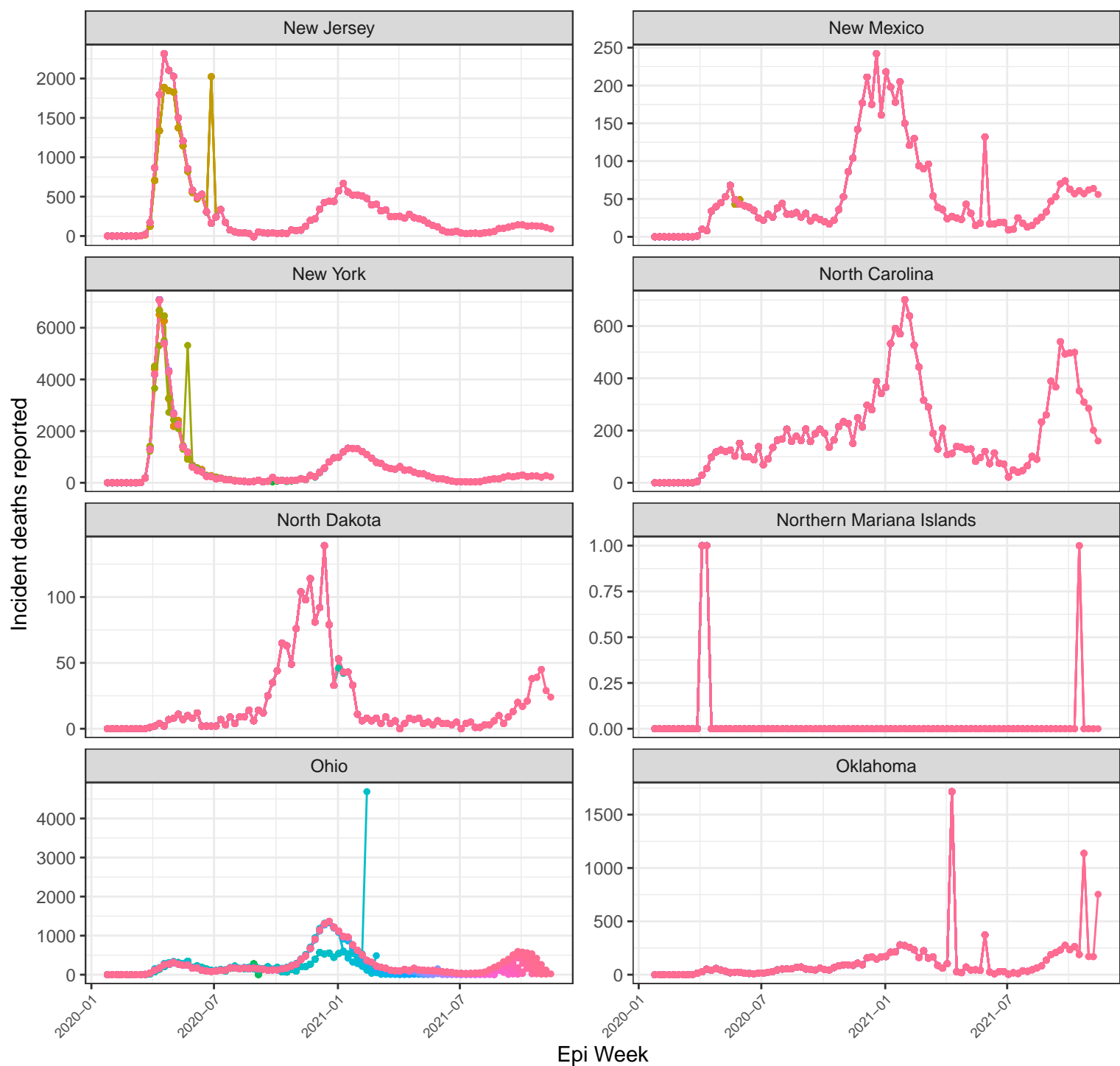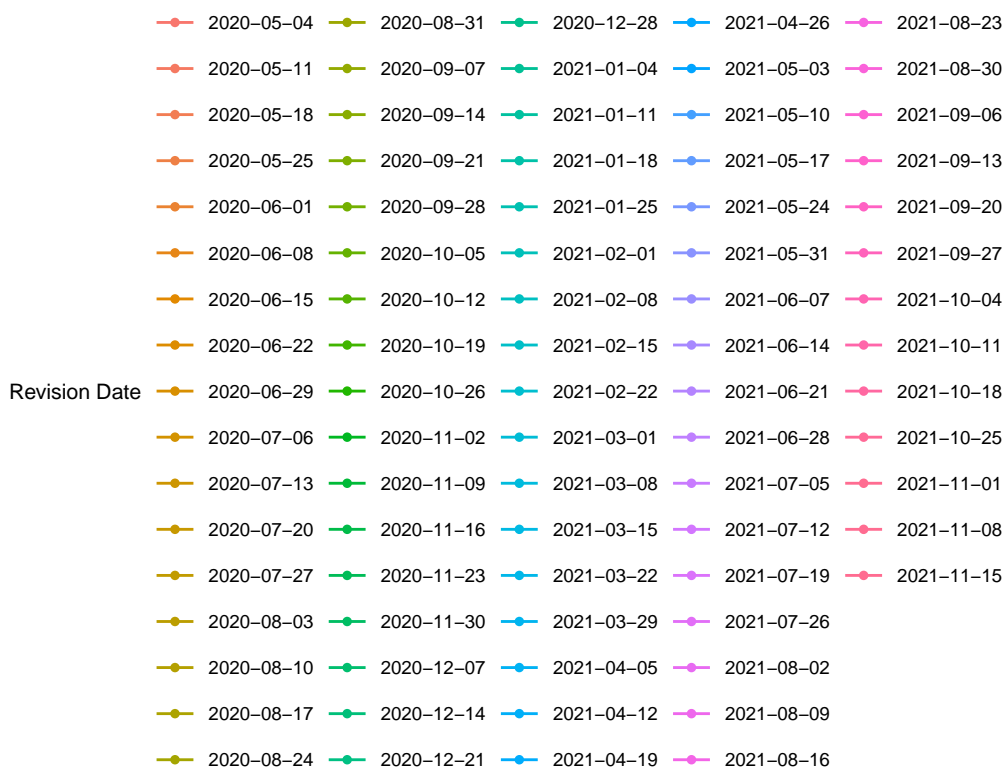

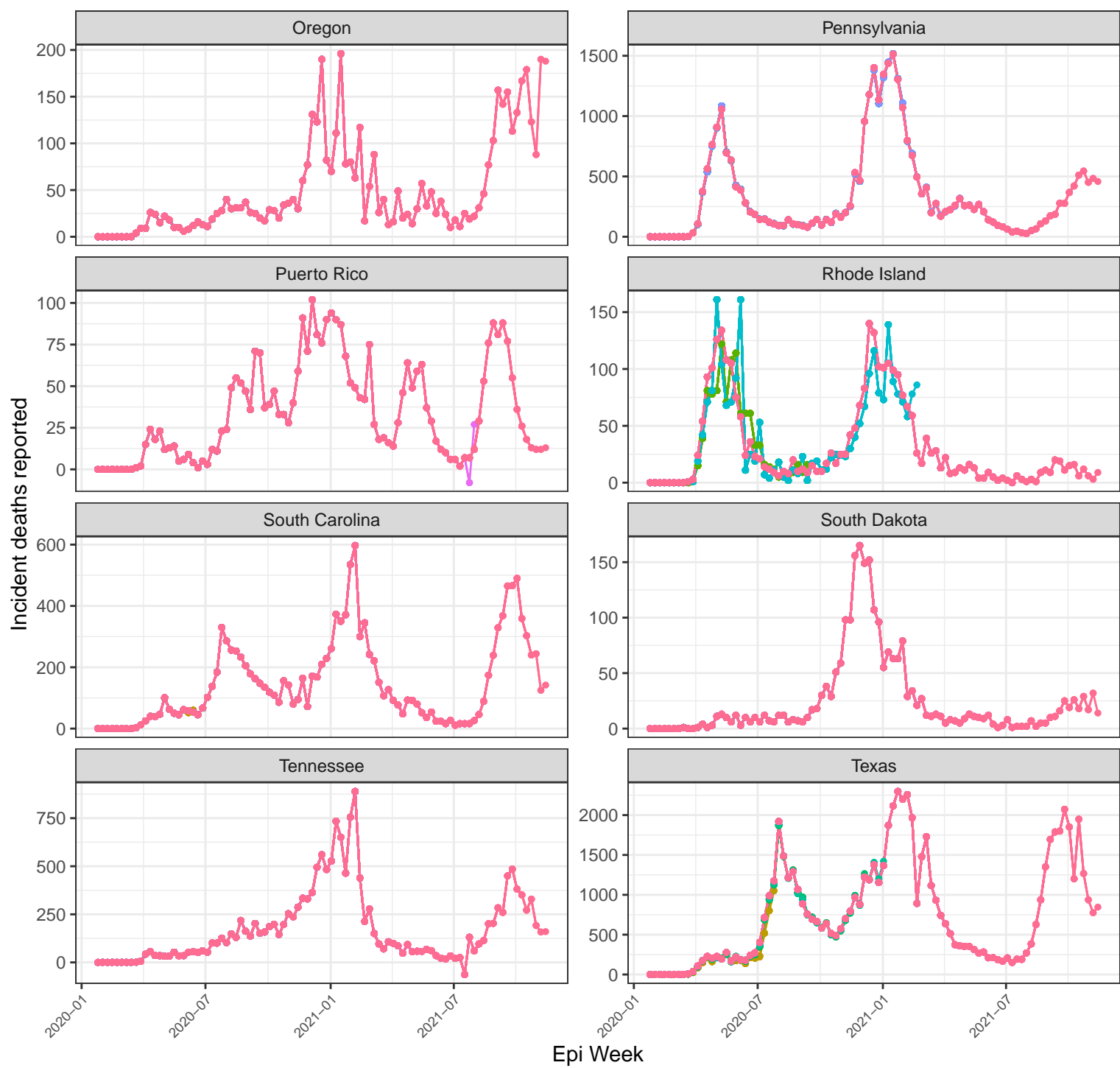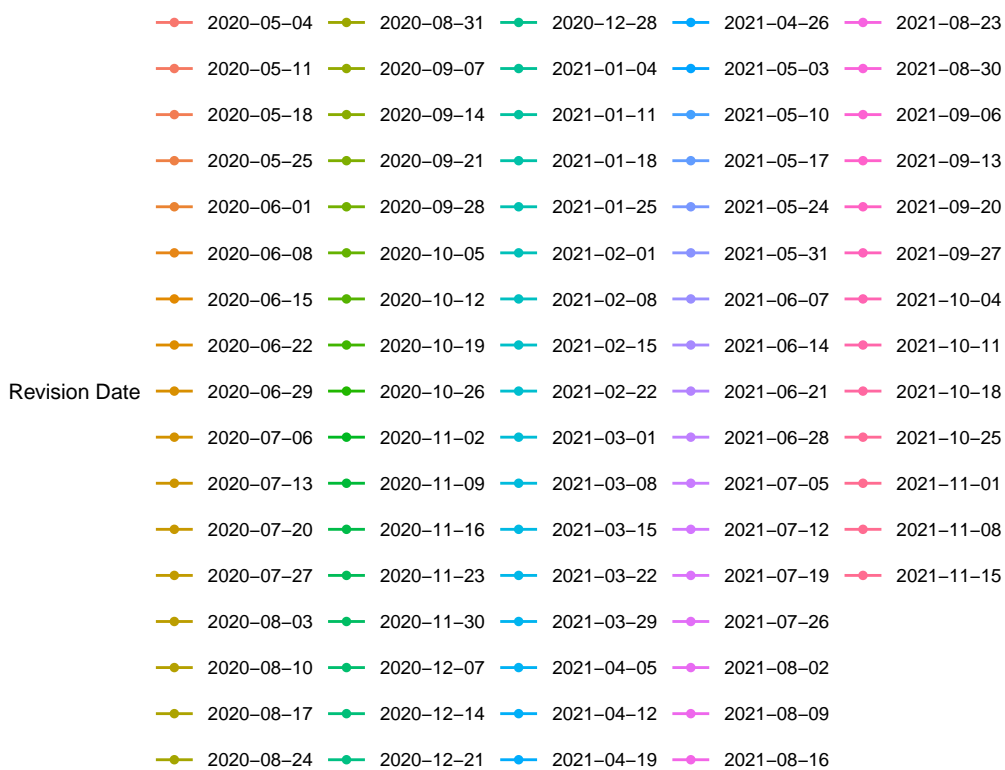

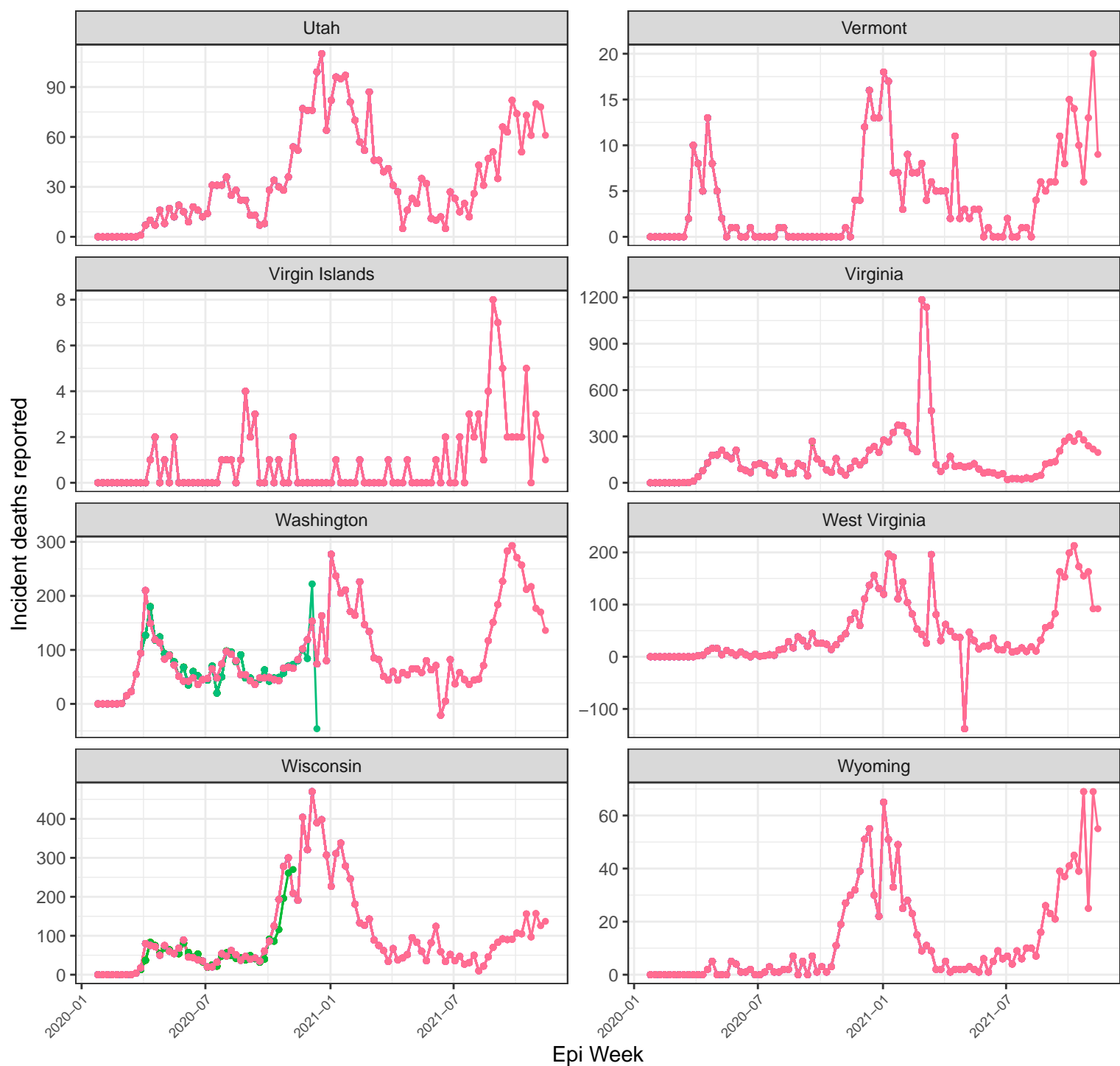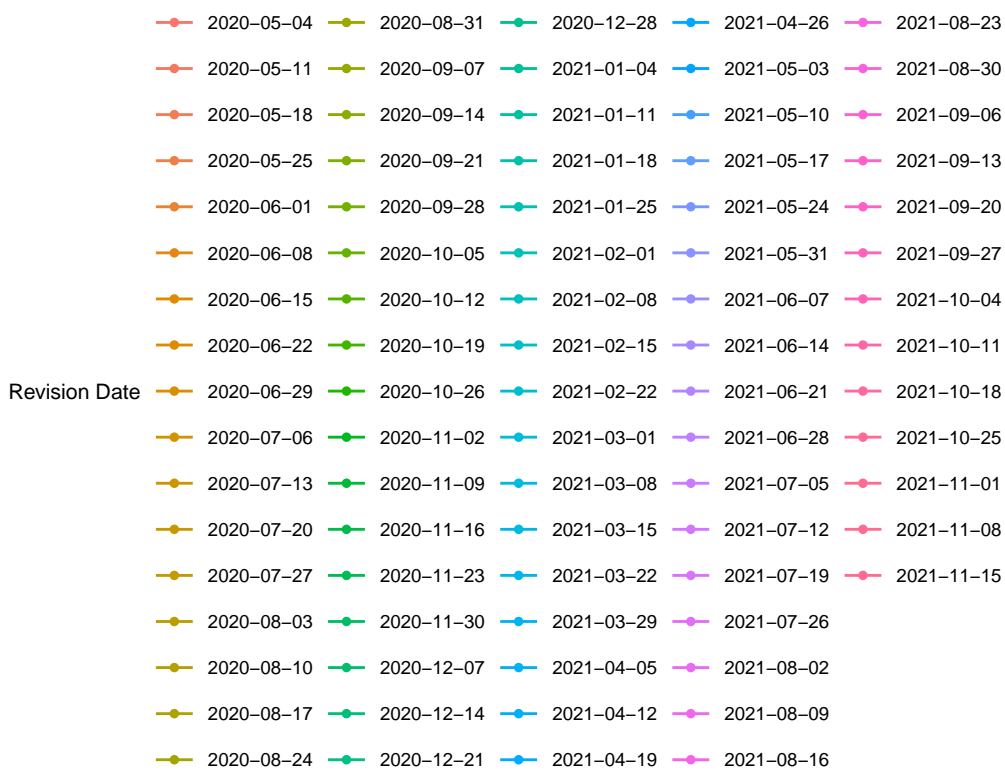

Supplement: Supplementary File [file pnas.2113561119.sd01.pdf]
